# Genomic approaches used to investigate an atypical outbreak of *Salmonella* Adjame

**DOI:** 10.1099/mgen.0.000248

**Published:** 2019-01-16

**Authors:** Marie Anne Chattaway, Nastassya Chandra, Anaïs Painset, Victoria Shah, Peter Lamb, Elsie Acheampong, Janice Lo, Bharat Patel, Lesley Larkin, Martin Sergeant, Martin Cormican, Eva Litrup, Paul Crook

**Affiliations:** ^1^​ Gastrointestinal Bacteria Reference Unit, Public Health England, London, UK; ^2^​ Field Epidemiology Service, Public Health England, London, UK; ^3^​ North West London Health Protection Team, Public Health England, London, UK; ^4^​ Public Health Laboratory, Public Health England, London, UK; ^5^​ Centre for Infectious Disease Surveillance and Control, Public Health England, London, UK; ^6^​ University of Warwick, Warwick, UK; ^7^​ School of Medicine, National University of Ireland Galway, Galway, Ireland; ^8^​ Bacterial Typing, Statens Serum Institute, Copenhagen, Denmark

**Keywords:** whole genome sequencing, surveillance, Salmonella, England, Adjame, Wales

## Abstract

In 2017, an outbreak of gastroenteritis in England attributed to *Salmonella* Adjame was detected and investigated. With the introduction of whole genome sequencing (WGS) for microbial typing, methods for comparing international outbreak data require evaluation. A case was defined as a person resident in England with a clinical sample from 1 June 2017 to 27 July 2017 from whom *S*. Adjame was isolated. Cases were interviewed and exposures analysed. Backward tracing of food provenance was undertaken. WGS was performed on isolates from cases and historical isolates and compared using Public Health England’s SnapperDB high-quality SNP pipeline and Enterobase’s *Salmonella* core genome multi-locus sequence typing (cgMLST) scheme. In total, 14 cases were identified. The majority were vegetarian, probably of South Asian descent, with a median age of 66.5 years with no recent international travel reported. Cases consumed a range of fresh food products including herbs and spices bought from South Asian grocers. Backward tracing did not identify a common source. WGS typing showed sub-clustering and considerable genetic variation across human samples. cgMLST allele-based analysis was comparable to SNP-derived phylogenetic analysis and clusters were defined using each method. Imported herbs or spices were suspected vehicles. The cases were linked in time and place but WGS showed marked heterogeneity, atypical of a point source *Salmonella* outbreak. The application of incorporating SNP or allelic differences into the case definition may not always be appropriate. With further validation, cgMLST could be used for international outbreak alerts when WGS analysis is being undertaken to facilitate comparison.

## Data Summary

FASTQ sequences were deposited in the NCBI Short Read Archive under the BioProject PRJNA248792 (https://www.ncbi.nlm.nih.gov/bioproject/?term=248792). Refer to Table S1 (available in the online version of this article) for SRR accession numbers.

**Outcome**

In this article we show how the use of whole genome sequencing for outbreak detection and use of SNP- or allele-based clustering methods as part of case definition may not always be appropriate in heterogeneous outbreaks or rare/unusual serovars involving small numbers of cases. Sequencing of the *Salmonella* Adjame outbreak strains produced a heterogeneous phylogeny showing multiple microbiological sub-clusters that were, in general, temporally and geographically linked. Contamination from an endemic source with a mixed population of strains could be expected to show a heterogeneous genealogy consistent with the hypothesized source of the contamination being imported herbs or spices from South Asia. We were also able to show correlation between SNP- and allele-based clustering methods in this outbreak and therefore recommend that core genome multi-locus sequence typing be used for international comparisons of data and sequence types shared via web-based communication platforms such as the Epidemic Intelligence Information System (EPIS).

## Introduction

Non-typhoidal *Salmonella* (NTS) causes an estimated 93.8 million illnesses globally each year with the majority of infections attributable to *Salmonella enterica* subsp. *enterica* [1]. In England and Wales, *S. enterica* serovars Enteritidis and Typhimurium are associated with the majority of NTS outbreaks and sporadic cases [51 % (4391/8691) of NTS isolates reported in 2017, Public Health England (PHE) unpublished data]. On occasion, rare serovars of *Salmonella* cause outbreaks and knowledge of previous likely vehicles of infection can help inform epidemiological investigations.

*S. enterica* subsp. *enterica* Serovar Adjame was first isolated from a clinical case from the Cote d’Ivoire and described in 1967 [2] but there have been no published reports regarding human infection or associated sources after this date. *S.* Adjame is a rare serovar in the UK. The first UK case was reported in 1993 and only 13 cases in total have been reported prior to 2016 (0–2 cases per year). *S*. Adjame has never been isolated from livestock or animal feed in Great Britain (Dr Robert Davies, Animal and Plant Health Agency, pers. comm.).

Public health experts can exchange information to assess whether current and emerging public health threats may be cross-border in the European Union (EU) using the Epidemic Intelligence Information System (EPIS) – a web-based communication platform (https://ecdc.europa.eu/en/threats-and-outbreaks/epidemic-intelligence). Traditional typing results such as PFGE or multilocus variable number of tandem repeats analysis (MLVA) profiles are used as a way for other countries to compare isolates to detect international outbreaks. Established phenotypic and molecular typing tools for *Salmonella* surveillance are steadily being replaced by whole genome sequencing (WGS) methods. However, there are a variety of methods for further characterization of sequence data being implemented. As a result, cross-border comparisons typically require the exchange of raw sequencing data so that each national reference centre can apply their own approach. Currently, there is no consensus regarding the format of a WGS profile that can be posted on public health alert systems with which other countries can easily compare their data.

At PHE, *Salmonella* surveillance is performed using a reference-based SNP detection pipeline. A set of tools, SnapperDB, is used to store bacterial variant data to facilitate reproducible and scalable analysis of bacterial populations [3]. Hierarchical, single linkage clustering of genetic distance is used to describe phylogenetic groups and generates a SNP address, which can be used to describe the relationship between isolates in a population at nucleotide resolution [4, 5]. An alternative approach to determine relatedness is to use a defined set of loci as a typing scheme. Enterobase is a public database with a core genome multilocus sequence type (cgMLST) scheme available for *Salmonellae* (http://enterobase.warwick.ac.uk/). The core genome sequence type (cgST) is defined by the combination of alleles at these loci and has the potential to be used as a complement to SNP typing and therefore could potentially be used for international comparison and alerting.

In August 2017, an increase in the number of *S*. Adjame isolates was reported initially at a local hospital, coinciding with an overall concomitant increase in the number of *S*. Adjame isolates reported by the Gastrointestinal Bacteria Reference Service at PHE. A local Incident Management Team (IMT) was established to investigate the increase in *S*. Adjame cases.

We describe the epidemiological and microbiological findings of the outbreak investigation and compare the microbial typing data derived from the routine PHE SNP-based pipeline and those derived from cgMLST.

## Methods

### Case ascertainment

The IMT coordinated the investigation. An outbreak case was defined as a person resident in England with a clinical sample from 1 June 2017 to 27 July 2017 from which *S*. Adjame was isolated. In England, all laboratories routinely send isolates of *Salmonella* from human clinical diagnostic samples to the PHE Gastrointestinal Bacterial Reference Unit (GBRU) for further characterization. Because all further characterization of *Salmonella* beyond the species level is carried out by GBRU, case finding was conducted by review of GBRU results.

An EPIS urgent enquiry was posted regarding the increase of *S.* Adjame cases in the UK. If a country responded having identified recent *S.* Adjame reports, further information was sought on the demographics and likely exposures, including travel to the UK. In addition, sequence data were requested for comparative analysis.

### Epidemiological analyses

A trawling questionnaire was used to interview cases, followed by a targeted questionnaire developed in response to information from initial cases, who were predominantly identified as South Asian and reported consuming foods typical of a South Asian diet. Questions included demographic and clinical information, recent travel history, food history and events attended in the 7 days preceding onset of illness. Specific details on where food products were purchased were requested. Data from questionnaires were extracted and descriptive analyses were undertaken in Stata-13 (Stata Statistical Software; StataCorp) and Microsoft Excel 2010 to describe cases and their exposures.

### Trace-back investigations

The Food Standards Agency and local authority environmental health teams undertook backward tracing of provenance for food items which were most commonly reported by cases, in order to identify common supply chains.

### Bacterial strains

All *Salmonella* isolates are routinely sequenced at PHE but not all *Salmonella* EBurst Groups (EBGs) are routinely SNP-typed and therefore further typing of this rare group was required. Strains selected for this study included *S*. Adjame isolates within and since the outbreak case definition time period, all viable historical *S*. Adjame isolates received at PHE, since its first report in 1993 and the originally isolated *S*. Adjame strain from Cote d’Ivoire provided by the Institute Pasteur. Sequence data derived for *S*. Adjame isolates available from other countries who responded to the EPIS alert were also included. In total, 31 *S*. Adjame were SNP-typed (20 UK isolates received in 2017, one reference strain, six historical UK isolates, two Irish isolates, one Danish isolate and the original 1966 Cote d’Ivoire isolate). Representative strains were deposited in the National Collection of Type Cultures (SRR5583198 - NCTC 14246, SRR6237100 - NCTC 14247, SRR6190984 - NCTC 14248).

### Whole genome sequencing

DNA extracted from *S.* Adjame isolates was sequenced by the PHE Genomics Development and Services Unit using the Illumina HiSeq 2500 platform in rapid run mode (-2×100 bp reads [6]). FASTQ reads were quality trimmed using Trimmomatic [7] with bases removed from the trailing end that fell below a PHRED score of 30. Sequence type (ST) assignment was performed using the Metric Orientated Sequence Typer (MOST) [8] available from https://github.com/phe-bioinformatics/MOST. MLST was undertaken using the MLST database described by Achtman *et al*. [9].

Due to the rarity of the serovar and lack of available sequenced isolates in the PHE database, phenotypic serology was used for the first three isolates of each ST to confirm the identification as described in the White–Kauffman–Le Minor scheme [10–12]. For additional isolates, the correlation between STs and phenotypic serology was used to confirm the Adjame serovar result [9].

### Analysis using Enterobase

Due to the rarity of the serovar, a PHE SNP database was not immediately available for analysis and cgMLST was used to assess the genetic similarity of the strains while an *S*. Adjame Snapper database [3] was created. Raw sequence data files of the isolates were uploaded to Enterobase (https://enterobase.warwick.ac.uk/) for cgMLST analysis to produce a cgST, which is automatic once the sequences have been uploaded. The first step in the process is assembly of the short reads using SPAdes and further downstream polishing [13]. The steps leading to the high-quality assembly are read pre-processing, trimmed, assembled, post-corrected and filtered according to the Enterobase Backend pipeline: QAssembly (http://enterobase.readthedocs.io/en/latest/pipelines/enterobase-pipelines.html). Assemblies that fail the quality control are not used for analysis. This produces a FASTA file whereby the assembly has been checked for base accuracy, contamination, etc. This FASTA file is then used to call cgMLST V2, which is based on 3002 genes that were chosen as being present in >98 % of a diverse reference set of 3144 genomes [13]. Allele sequence and loci information for the scheme can be downloaded from Enterobase, http://enterobase.warwick.ac.uk/species/senterica/download_data. Alleles for each locus are identified in the assembly and fragmented, duplicated or poor quality alleles are assigned negative values and missing alleles 0. Otherwise each new allele is assigned a new sequential number. As with conventional seven-gene MLST, each unique combination of alleles (3002 in this case) is assigned a specific ST. Similar but non-identical strains (those that did not have identical cgSTs) were identified in Enterobase by using the facility that allows searching for strains which can differ up to a specified number of alleles. The minimum spanning tree was created in Enterobase using the MSTreeV2 algorithm [14].

### SNP typing

*S.* Adjame isolates were subtyped using SNP analysis [3]. Whole genome SNP analysis was carried out by mapping the strains of interest against a multi-contig reference genome from the same serovar (for this analysis SRR5583198 was used as a reference). This reference is an assembly of the reads performed by using SPAdes v3.8.0 [15]. Isolates were mapped against the reference genome using BWA mem [16]. The Sequence Alignment Map output from BWA was sorted and indexed to produce a Binary Alignment Map (BAM) using Samtools [17]. GATK2 [18] was used to create a Variant Call Format (VCF) file from each of the BAMs, which were further parsed to extract only SNP positions which were of high quality (MQ >30, DP >10, Variant Ratio >0.9) using SnapperDB [3] (software available on https://github.com/phe-bioinformatics/snapperdb). Sequences with coverage above 30 were added to the database. Pseudosequences of polymorphic positions were used to create maximum-likelihood trees using RaxML v8.2.8 with the gamma model of rate heterogeneity and 1000 bootstraps undertaken [19].

## RESULTS

### Epidemiology

Fourteen cases met the outbreak case definition, including 11 confirmed cases of *S*. Adjame (serotype 13,23:r:1,6) in London and one case each in the following regions of England: the South East, East of England and the South West. Additionally, there were five reports of *S.* Adjame from human samples in England between March and May 2017, and one report in October 2017 which did not meet the outbreak case definition and therefore were not included in the epidemiological investigation.

**Fig. 1.**
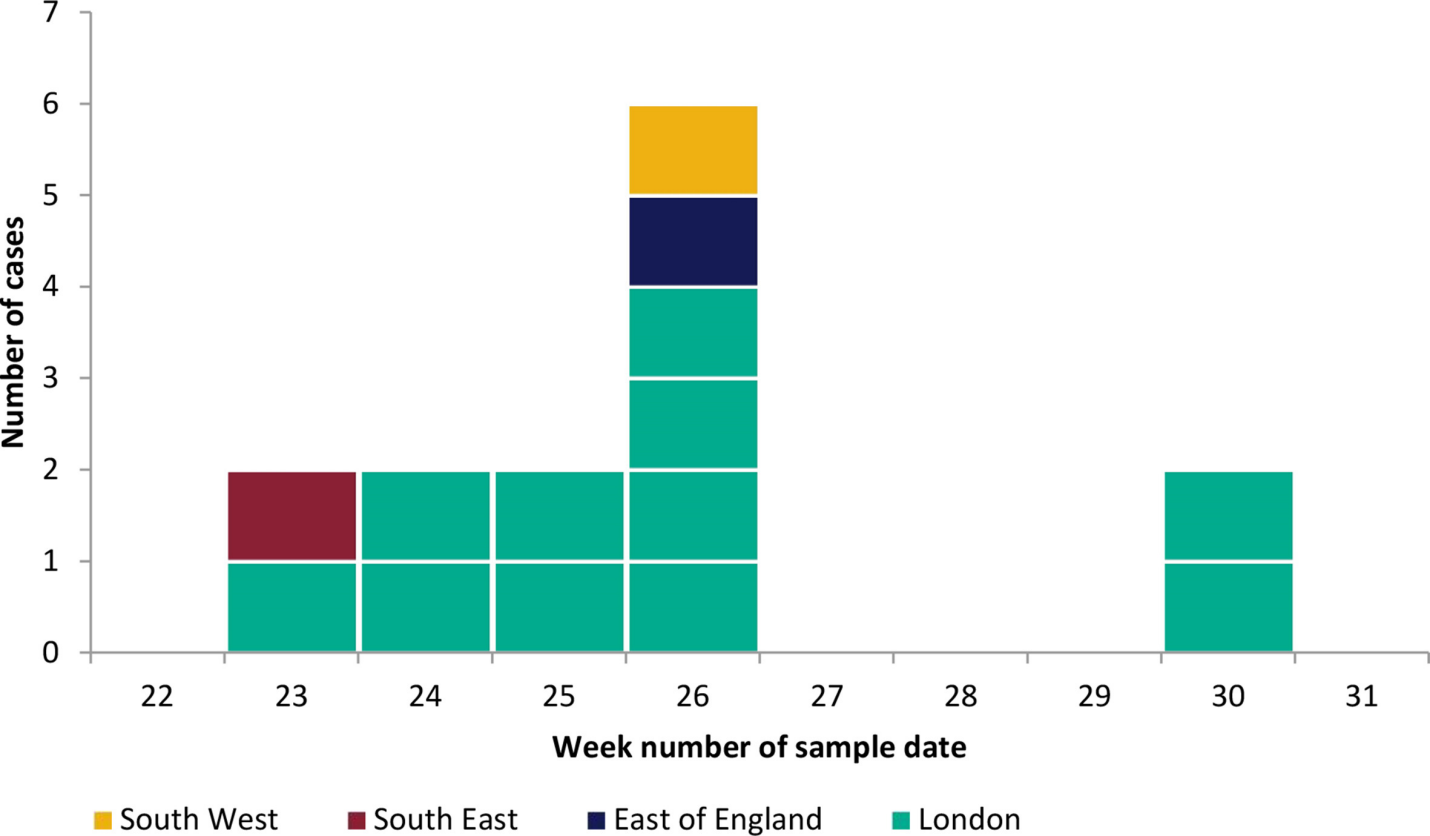
Sample date of *Salmonella* Adjame outbreak cases by calendar week and by resident area, June to July 2017. Week 22 = 28 May 2017 to 3 June 2017.

Sample dates for the outbreak cases investigated ranged from 5 June to 26 July 2017 (Fig. 1). Eight cases were male and six cases were female. The median age of cases was 66.5 years (range 3–85 years), presenting with a range of clinical symptoms (Table 1). All but one of the cases were considered to be of South Asian descent based on a review of names.

**Table 1.** Characteristics, clinical information and phylogeny of *Salmonella* Adjame cases, from London, South East, South West and East of England, June to July 2017

Case information					Clinical information				*Salmonella* Adjame subtyping data	
Case number	Accession no.	Area of England	Specific questionnaire complete	Onset date*	Duration of illness (days)	Hospitalized	Duration of hospital stay (days)	Symptoms	ST	Sub-cluster (Figs 2 and 3)
1	SRR6237100	London	Yes	04/06/2017	10	No	–	Diarrhoea; nausea; vomiting, fever, dizziness	3929	2-Green
2	SRR6234003	London	Yes	08/06/2017	10	Yes	4	Diarrhoea; nausea	3929	2-Green
3	SRR6233939	London	Yes	15/06/2017	–	No	–	Diarrhoea; abdominal pain	3929	2-Green
4	SRR6233881	London	Yes	23/06/2017	4	No	–	Diarrhoea	4023	3-Red
5	SRR6193063	London	Yes	21/06/2017	>14	No	–	Diarrhoea; abdominal pain; fever	4023	3-Red
6	SRR6233875	London	Yes	23/06/2017	3	Yes	1	Diarrhoea; abdominal pain, co-infection with	4023	None
								*S*. Agbeni		
7	SRR6192965	London	No	25/06/2017	–	–	–	–	4023	3-Red
8	SRR6191380	London	Yes	24/06/2017	7	No	–	Diarrhoea; abdominal pain; fever	4023	3-Red
9	SRR6190984	London	Yes	29/06/2017	9	Yes	9	Diarrhoea; vomiting; rigors	4023	3-Red
10	SRR6191331	London	Yes	17/07/2017	>14	Yes	7	Diarrhoea; abdominal pain; headache	4023	3-Red
11	SRR6190990	London	Yes	30/05/2017	>14	No	–	Diarrhoea; blood in stools; vomiting; abdominal pain; fever	4023	3-Red
12	SRR6193034	East of England	Yes	24/06/2017	14	No	–	Diarrhoea; nausea; vomiting; abdominal pain; fever; headache; mucus	4023	3-Red
13	SRR6191319	South East	No	–	–	–	–	–	3929	2-Green
14	SRR6191533	South West	No	10/06/2017	3	No	–	Diarrhoea; nausea; vomiting; abdominal pain; fever; headache; feeling weak	3929	2-Green

*Some onset dates are estimated.

Clinical information was collected for 12 cases, all of whom had reported diarrhoea and the majority reported abdominal pain (*n*=8) (Table 1). Four cases reported being admitted to hospital (1–9 days duration) and one case visited the emergency room. Three cases stated that they were still ill at the time of being interviewed; the eight cases who had recovered and for whom information was available had a median duration of illness of 8 days (range 3–14 days). No deaths were reported. One case had a co-infection with *S*. Agbeni, isolated from a faecal specimen taken 2 days prior to the faecal specimen from which the *S*. Adjame isolate was obtained.

Trawling questionnaires were completed for 11 cases, although two cases did not complete all sections with regard to food exposures. Ten cases completed information on herbs and spices and nine completed information on fresh products. Seven cases considered themselves as vegetarian (two also ate fish products). Of the four who were not vegetarian, one case did not eat chicken or beef.

No cases reported international travel in the 7 days prior to illness. The East of England case mentioned travel to North West London, where the majority of cases were resident, on a regular basis. Three cases reported eating takeaway meals from different branches of the same restaurant chain, although this was not further investigated because it only accounted for 27 % of the interviewed cases with limited information on what had been consumed. Two cases reported eating at restaurants prior to becoming ill. No cases reported attending any events 7 days prior to becoming ill.

We identified grocery stores and food items common to many of the cases. Of particular interest were a number of South Asian grocers in North West London (identified as grocers A–F) that sold a range of fresh fruit and vegetables, herbs, spices and food items commonly used in South Asian cooking. Some of these grocers had a number of different branches. Eight cases mentioned buying products from grocer A, four from B, three from C, two from D and E, and one from F. The East of England case regularly visited the North West London area and was one of the cases reporting purchasing products from grocer A and grocer B.

The food exposures most commonly reported by cases were salad, fruit, vegetables, herbs and spices. Pepper and turmeric were the most commonly used spice/herb exposures (9/10) closely followed by chilli powder (8/10), fresh coriander (8/10), cinnamon (powder/bark, 7/10) and coriander seeds/powder (6/10). In addition, the following were frequently reported; Lettuce, tomatoes, onions and bananas (all 8/9), followed by cucumber (7/9), spinach (6/9), peppers (6/9), apples (6/9), oranges (5/9) and grapes (5/9).

We identified three brands of spices commonly consumed by cases (brands X, Y and Z). We calculated that 10/11 cases had a likelihood of being exposed to X, 8/11 to Y and 7/11 to Z. Three of the 11 cases had a chance of being exposed to a range of other brands of spices sold at the stores that they reported visiting.

The results from the trace back of the common spice brands and other common food items (lettuce, tomatoes, onions and bananas) that were reported being purchased from the South Asian grocers showed that there were multiple possible sources for each component and no common suppliers were identified across all the grocers. Grocers B and D are also wholesalers and supplied to numerous other stores and businesses. Grocer D supplied to major supermarkets. The tracing identified that for many of the products the country of origin and suppliers change daily, although some products of interest were sourced outside Europe, particularly spices which were frequently sourced from South Asia spice farms. Where information was available, lettuce, tomatoes and other fresh fruit/vegetables tended to be sourced within the UK or Europe. In response to the EPIS urgent enquiry, four countries reported *S.* Adjame cases. Italy reported an *S.* Adjame case identified in June 2017, but there was no further information available. Germany reported two cases, with onset dates of 9 June (male, 27 years) and 20 July (female, 61 years), with no history of travel prior to illness. Between 2001 and 2016 only seven *S.* Adjame infections had been reported in Germany. Ireland had not previously reported *S.* Adjame infections until June 2017 when two reports were received (male and residents in Dublin), one case was of South Asian decent and one case reported recent travel, but there was no further information available. Denmark did not report any *S.* Adjame infections in 2015–2016, although they reported a Sri Lankan male with a sample date of May 2017, who had not travelled prior to illness but had reported purchasing and eating fruits, vegetables and spices bought from a South Asian grocery store in Denmark. It is unknown whether these individuals had the same food exposures as the UK cases as the same questionnaire as used for the UK cases was not used to interview the international cases and we were unable to identify likely vehicles of infection. Austria, Cyprus, Estonia, France, Luxembourg, the Netherlands, Slovenia and Sweden reported no recent reports of *S.* Adjame. In Georgia and Norway, *S.* Adjame had never been detected.

### Genomic analysis

The antigenic profile by phenotypic serology was 13,23:r:1,6 and the identification of the strains was confirmed as *S.* Adjame by WGS. All strains fell into one EBurst Group (EBG421) comprising two STs: ST4023 and ST3929.

**Fig. 2.**
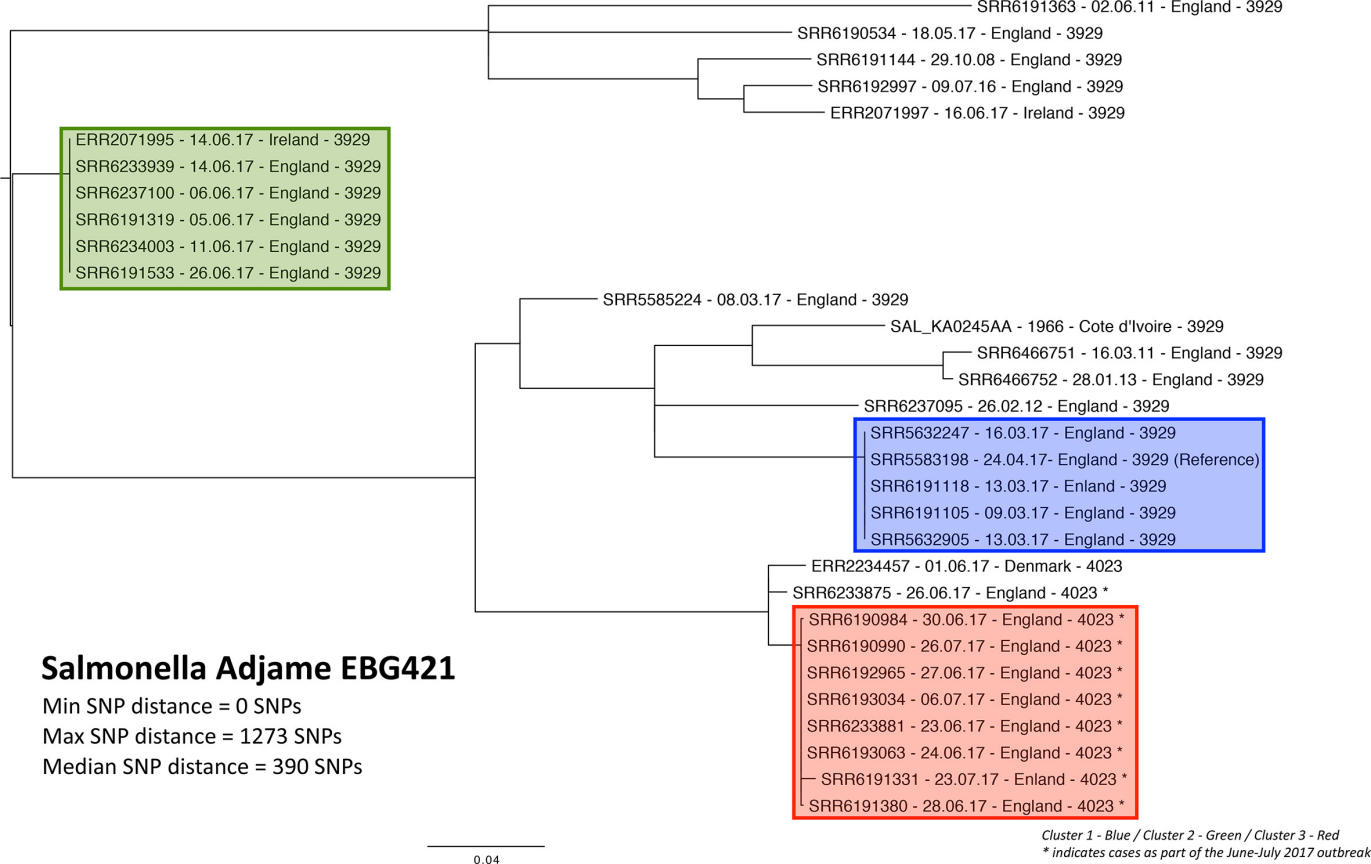
Phylogeny of *Salmonella* Adjame strains. *Cases as part of the June–July 2017 outbreak.

SNP clustering of all isolates indicated three genetically distinct clusters as well as single strains falling outside these clusters across the phylogeny (Table 1, Fig. 2). In total, 13/14 cases fell into two clusters (Red and Green) with one strain not genetically related to any of the three clusters. Cluster 1 (blue cluster) comprised five strains with zero SNP distance isolated in March/April 2017, cluster 2 (green cluster) comprised six strains with zero SNP distance (including one strain from Ireland) predominately isolated at the beginning of June 2017 and the third cluster (red cluster) comprised eight strains isolated in late June/early July 2017 (min. SNP: 0, max. SNP: 9). The maximum SNP distance between cluster 1 and 2 was 449 SNPs, between 1 and 3 692 SNPs, and between 2 and 3 410 SNPs. The remaining five strains from 2017 (UK=3, Ireland=1 and Denmark=1) were not closely genetically related to the clusters or each other (between 26 and 1236 SNPs from any of the main clusters) (Fig. 2, Table S1).

**Fig. 3.**
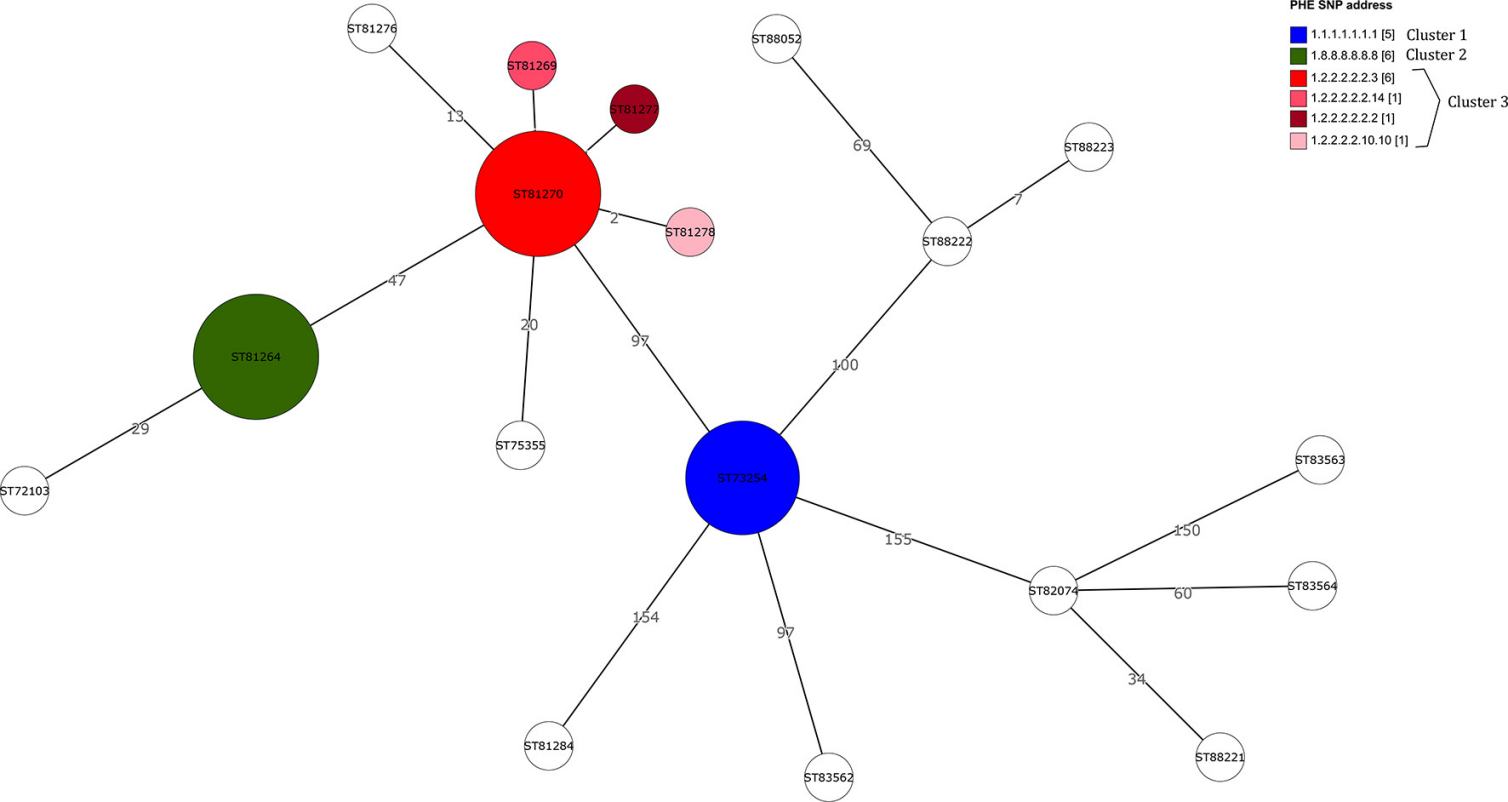
Grape Tree comparing SNP address versus cgMLST Enterobase analysis.

The clusters defined by the cgMLST analysis reflected the same clustering pattern found with the SNP clustering (Fig. 3, Table S1). There was zero allele difference between the isolates falling into cluster 1 (blue cluster), zero allele difference between isolates in cluster 2 (green cluster) and between zero and two alleles difference for the third cluster (red cluster). The maximum distance between cluster 1 and 2 was 101 alleles, between 2 and 3  99 alleles, and between 1 and 3   49 alleles.

The cgST (allele-based method) and the SNP address (SNP-based method) shows the same tendency among the isolates, whereby an increasing allele difference was associated with an increase in SNP difference between strains. For the isolates outside of the clusters and where the difference between strains was between 26 and 1236 SNPs, the cgST differed between 13 and 155 alleles between the strains (Fig. 3).

Across the 11 cases where a trawling questionnaire was completed, there are five known different SNP addresses. Three cases share SNP 1.8.8.8.8.8.8 and five cases share 1.2.2.2.2.2.3. Of the three cases with SNP 1.8.8.8.8.8.8, we have grocer information for two, both of whom shopped at grocer C. For the cases that share SNP 1.2.2.2.2.2.3, four of five cases shopped at grocer A and the same cases also shopped at grocer B; the fifth case with this SNP address shopped at grocer E (Table 2).

**Table 2.** SNP address of strains in association with shopping at a South Asian grocer

Case number	SNP address from GDW	South Asian grocer
1	1.8.8.8.8.8.8	C
2	1.8.8.8.8.8.8	A, C
3	1.8.8.8.8.8.8	Information not provided
4	1.2.2.2.2.2.3	A, B, D
5	1.2.2.2.2.2.3	E
6	1.2.2.7.7.7.7	A, B, D
8	1.2.2.2.2.2.14	A, E
9	1.2.2.2.2.2.2 or 1.2.2.2.2.2.3	A,B
10	1.2.2.2.2.10.10	C, F
11	1.2.2.2.2.2.3	A, B
12	1.2.2.2.2.2.3	A, B

GDW, gastrointestinal data warehouse.

## Discussion

We describe a small outbreak of *S.* Adjame, with marked genetic diversity. Outbreak cases were mainly older adults, probably of South Asian descent, vegetarian, and living in a geographically restricted area of North West London. Although a specific food vehicle associated with this outbreak was not identified, the hypothesis was that the most likely vehicle(s) of infection were herbs and spices obtained at local speciality grocery stores. Little is known about *S.* Adjame. This was the first outbreak of *S.* Adjame described since its isolation in 1966 from West Africa [2].

Our hypothesis arose from the following evidence: herbs and spices were the most commonly reported food item eaten and these were mainly obtained at local South Asian grocery stores, the small number of cases, geographical clustering, the case demographics (13/14 within a similar ethnic grouping) and the largely South Asian diet. The vegetarian diet of many of the cases allowed foods commonly associated with *S. enterica* outbreaks such as meat and poultry products to be excluded. While other common food items were frequently mentioned by the cases such as salad, fruits and vegetables, the relatively wide temporal spread of cases within clusters may indicate a product with a longer shelf life than would be expected for a fresh product. However, ongoing introduction of a persistently contaminated crop with staggered harvesting times, freezing of a fresh herb product and cross-contamination at the retail level cannot be excluded as a possible contributory factor to a longer than expected timeline. Analytical epidemiological investigation was not undertaken and it was not feasible to conduct product sampling due to the large number of potential retailers and suspect food items identified. The low likelihood that a contaminated product remained on sale at the time of the investigation was also a factor. In addition, products bought by cases being unidentifiable due to removal of packaging and products already being consumed by cases as well as products being mixed together (as described in the questionnaires) also prevented product sampling.

A hypothesis of a dried herb or spice being the vehicle of infection is plausible. *S. enterica* has the ability to survive in non-host environments and to resist dry conditions for extended periods, allowing for dried spices to be a potential vehicle of viable bacteria [20]. Lehmacher *et al.* found *S. enterica* could survive for at least 8 months in samples of dried paprika [21]. In low-moisture foods, such as spices, salmonellae are desiccated, which has been shown to increase the organism’s tolerance to heat [22]. As a result, cooking of spices may not prevent infection if added at the end of the cooking procedure and therefore not thoroughly cooked. In an average ‘Western’ diet, spices tend to be consumed in small quantities [23] which would limit the dose of *S. enterica* the consumer is exposed to, but the cases identified in this outbreak reported having a largely South Asian diet of which they described using a range of spices in most of their meals. Many of the herbs and spices consumed by the cases are subject to a known high risk of *S. enterica* contamination during production [23]. Human or animal activity may introduce faecal contamination into the herb/spice growing environment, directly contaminating the products. Furthermore, herbs and spices are commonly dried following harvesting in the open environment. This drying phase allows for further direct contamination due to possible exposure to vermin [23] and potentially other wildlife.

The hypothesis was that the vehicle of infection in this outbreak was likely to be a product imported outside of the EU/European Economic Area (EEA), and in an area with less robust surveillance systems for *S. enterica*. This was supported by the vehicles of interest (herbs/spices) often being sourced from South Asia and the rarity of *S.* Adjame in the EU/EEA. However, there is no robust evidence from the food trace back that was undertaken, which was challenging due to the number of potential food products of interest and complex supply chains. Between January 2014 and December 2017 there have been 110 notifications to the European Commission Rapid Alert System for Food and Feed (RASFF) of *S. enterica* contamination in imported herbs and spices [24], indicating this type of contamination is an issue on an international scale.

The phylogenetic diversity of the strains also lends support to the vehicle of infection being imported from an area, outside the EU, where the strain is persistently present in a source environment or host population where strains have had time to diversify. With the multiple potential points of contamination that spices could be exposed to during the production and importing process, a mixed population of *S.* Adjame responsible for an outbreak as shown by the genomic analysis in this study (Figs 2 and 3) is possible. However, it is also possible, considering the heterogeneity of the strains of *S*. Agama isolated, that multiple sources of contamination leading to more than one ‘outbreak’ were involved in this incident, but the investigators consider it most likely, based on the epidemiological information derived from cases and the food supply chain investigations, that this was probably one outbreak with multiple strain contamination at the source of related food vehicles. We were not able to clarify this further due to the small number of cases involved and the short duration of the outbreak.

With multiple potential routes of contamination for a product, or specifically contamination via human sewage, it could be expected that a variety of different serovars would be detected in patients following exposure to the contaminated food vehicle. This can explain how imported food sources such as spice can be contaminated with multiple *S. enterica* serovars as shown in previous studies [20], ultimately resulting in multi-serotype/species outbreaks. In the outbreak we describe, one patient was found to have co-infection with another rare serovar – *S*. Agbeni (Table 1). This finding was by chance as, untypically, multiple faecal specimens were collected from this patient and thus more than one isolate was sequenced. Usually, with a single specimen only one pick of *Salmonella* is taken from culture for sequencing. Therefore, this could suggest that other co-infections of *Salmonella* could have been present in other cases. To have the majority of the same serovar in this outbreak suggests perhaps that there was some remote reservoir where this strain has been circulating for a very long time accruing considerable diversity. The product could then have become contaminated by that source (or a number of ‘source populations’ that had acquired the common ancestor from the same place and then evolved independently of one another but in close geographical proximity). Alternatively, other co-infections may have existed and were just not detected.

The rarity of the serovar and the lack of sufficient isolates for phylogenetic comparison meant that an atypical approach was taken in this outbreak investigation compared to the usual approach taken by PHE since implementation of routine WGS for *Salmonella*. Specifically in this instance, all cases of this serovar in a specified time period were included in the outbreak definition and were investigated despite considerable genetic variation between the strains falling into two distinct clusters reported in June and July 2017 (Cluster 2, Green; Cluster 3, Red; Figs 2 and 3). The rationale for this was that the epidemiological link of a rare serovar occurring in a local geographical area in a short time was indicative of an outbreak. Due to the rarity of the strain and the initial lack of an SNP database, serotyping and cgMLST analysis was also carried out. WGS has revolutionized the way outbreaks are detected and has proven to be sensitive and specific in defining cases during outbreak investigations as isolates from cases within *Salmonella* outbreaks are commonly genetically homogenous and cluster monophyletically [5]. However, increased genetic variation has been observed for outbreaks where there is long-term strain persistence in the host population over an extended time-frame or a multi-strain population that has been independently introduced into the food chain [25, 26]. However, in the outbreak we describe, the phylogeny of cases and historical isolates showed great heterogeneity with three paraphyletic clusters including cluster 1 which occurred before the cases defined in this outbreak. The cases in cluster 1 were temporally linked as well as other genetically distant strains within the same time period in the same geographical area and therefore may have been part of the same outbreak (Fig. 2). An alternative explanation, namely that there are really two distinct outbreaks, during the case definition, but which happen to overlap slightly in time, although unlikely, cannot be ruled out. In this incident, the heterogeneity of the strains of *S*. Agama could be considered to indicate multiple sources of contamination and therefore we were dealing with more than one ‘outbreak’, but the epidemiological information derived from cases and the food supply chain investigations appeared to indicate this was probably one outbreak with multiple strain contamination at source of a specific food vehicle. We were not able to clarify this situation due to the small number of cases involved and the short duration of the outbreak(s). We consider that it is possible in this scenario for quite different variants to be isolated from individuals infected from the same batch of product. This is important because it makes the concept of using inflexible thresholds of genetic relatedness (by SNP or cgMLST) that excludes a link between cases potentially unsound. This means that advances in molecular biology enhance the potential of microbiology to complement strong epidemiology but should never replace the epidemiological association of ‘time–person–place’ in an uncommon event. This outbreak was an example of how genetic differences, such allele or SNP differences, may not always be appropriate to include as part of a case definition. When genetic thresholds are used they need to be flexible to accommodate different epidemiological contexts.

In contrast to the typical approach taken by PHE, SNP or allelic differences were not used to define the outbreak and limit the case definition microbiologically to a more granular level than the serovar level. This was due to the fact that although cases did not fit into a traditional ‘5 or 10 SNP cluster’, the cases were clearly epidemiologically linked. This included temporal links, geographical and population demographic links between the majority of the cases as well as the commonality in food exposures. In addition, the locations of purchase determined during the epidemiological investigations were also linked and therefore important to follow up all cases.

Given the processes of herb and spice production, including consolidation from multiple small producers, contamination could occur at multiple time points and/or multiple locations. This could then lead to the potential introduction of very distantly related members of the same serovar or of multiple serovars into a single batch of product.

WGS analysis methods are replacing traditional typing methods such as MLVAor PFGE, but as yet there is no single internationally agreed way of describing WGS profiles (analogous to MLVA of PFGE profiles) to enable global comparability. There are networks available for comparing WGS data such as Genome Trakr [27], and PulseNet International (PNI) in which a vision has been developedto standardize strain nomenclature [28], but it will take time for a harmonized method to be agreed upon. Currently, with suspected international outbreaks, sequence data are usually shared between organizations and incorporated into local analytical pipelines with the resulting phylogenies compared. This is more time consuming than just comparing profiles. However, cgMLST is universal, computationally cheap and fits in with the harmonized WGS vision of PNI [28]. cgMLST analysis of the strains within this atypical and genetically diverse outbreak showed that the allele differences of the strains correlated with the SNP analysis method used by PHE. cgMLST using the publicly available Enterobase database has the potential to be a standard test for generating cgST information to share on platforms such as EPIS for international comparison. With further validation using a variety of *Salmonella* serovars and isolates from previously defined outbreaks, cgMLST analysis could be used by reference laboratories for the sharing of relevant outbreak data between organizations. Moving forward, a central allele database accessible to all would enable organizations to incorporate the method into their local pipelines routinely.

### Conclusion

We describe an outbreak of *S.* Adjame mainly in South Asian vegetarians living in North West London. Although imported produce, probably herbs or spices, were suspected vehicles, there was only descriptive epidemiological evidence supporting this. While the cases of *S.* Adjame were linked epidemiologically in time, place and person, WGS showed marked heterogeneity, indicating a multi-strain outbreak, atypical of the *S. enterica* outbreaks PHE usually investigates. This indicates that while genomic criteria for inclusion of isolates in a cluster for investigation are of value, great care is needed in applying the concept of genomic-based criteria for exclusion of isolates, particularly where classical epidemiological evidence suggests an association. The interpretation of genomic analysis in regards to *S. enterica* outbreak investigations needs to be adaptable to the particular circumstances of the outbreak in terms of case definition. cgMLST is a potential method for outbreak comparisons but further validation is required

## Data bibliography

1. Dallman T. J., Ashton P. A., Jenkins C., Grant K. NCBI Short Read Archive PRJNA248792 (2014).

## Supplementary Data

Supplementary File 1
