# Supplementary material for: Genomic approaches used to investigate an atypical outbreak of Salmonella Adjame
Source: Microb Genom. 2019 Jan 16;5(1):e000248. doi: 10.1099/mgen.0.000248 (PMC6412060; doi:10.1099/mgen.0.000248)
Supplement: Supplementary File 1 [file mgen-5-248-s001.pdf]

Supplementary Table 1 Reference and genomic information for reports of *Salmonella* Adjame

Reference	Accession Numbers	Enterobase reference	Sample Date	Foreign Travel	Organism Identified	MLST	EBG	cgMLST	SNP Address
353868	SRR6191105	SAL_KA8931AA	09/03/2017	N	*Salmonella* Adjame	3929	421	71258	1.1.1.1.1.1.1
353918	SRR5585224	SAL_JA5441AA	08/03/2017	N	*Salmonella* Adjame	3929	421	72103	1.12.12.12.12.12.12
355050	SRR5632905	SAL_JA8036AA	13/03/2017	Unknown	*Salmonella* Adjame	3929	421	71258	1.1.1.1.1.1.1
356310	SRR5632247	SAL_KA8742AA	16/03/2017	Unknown	*Salmonella* Adjame	3929	421	73254	1.1.1.1.1.1.1
357971	SRR6191118	SAL_KA8743AA	13/03/2017	N	*Salmonella* Adjame	3929	421	71258	1.1.1.1.1.1.1
374589	SRR6190534	SAL_KA8744AA	18/05/2017	Unknown	*Salmonella* Adjame	3929	421	81284	5.13.13.13.13.13.13
381330	SRR6191319*	SAL_KA8746AA	05/06/2017	N	*Salmonella* Adjame	3929	421	81264	1.8.8.8.8.8.8
385774	SRR6193063	SAL_KA8747AA	24/06/2017	Unknown	*Salmonella* Adjame	4023	421	81270	1.2.2.2.2.2.3
387049	SRR6192965	SAL_KA8749AA	27/06/2017	N	*Salmonella* Adjame	4023	421	81270	1.2.2.2.2.2.3
387137	SRR6233881*	SAL_KA8750AA	23/06/2017	Unknown	*Salmonella* Adjame	4023	421	81270	1.2.2.2.2.2.3
387215	SRR6191533*	SAL_KA8751AA	26/06/2017	Unknown	*Salmonella* Adjame	3929	421	81264	1.8.8.8.8.8.8
387507	SRR6237100*	SAL_KA8755AA	06/06/2017	India[#]	*Salmonella* Adjame	3929	421	81264	1.8.8.8.8.8.8
387511	SRR6234003*	SAL_KA8754AA	11/06/2017	Unknown	*Salmonella* Adjame	3929	421	81264	1.8.8.8.8.8.8
388665	SRR6191380*	SAL_KA8752AA	28/06/2017	Unknown	*Salmonella* Adjame	4023	421	81269	1.2.2.2.2.2.14
388789	SRR6190984*	SAL_KA8757AA	30/06/2017	Unknown	*Salmonella* Adjame	4023	421	81277	1.2.2.2.2.2.2
389598	SRR6233875	SAL_KA8756AA	26/06/2017	N	*Salmonella* Adjame	4023	421	81276	1.2.2.7.7.7.7
389724	SRR6193034*	SAL_KA8760AA	28/06/2017	Unknown	*Salmonella* Adjame	4023	421	81270	1.2.2.2.2.2.3
399284	SRR6191331*	SAL_KA8758AA	23/07/2017	Unknown	*Salmonella* Adjame	4023	421	81278	1.2.2.2.2.10.10
400321	SRR6190990*	SAL_KA8759AA	26/07/2017	Unknown	*Salmonella* Adjame	4023	421	81270	1.2.2.2.2.2.3
409960	SRR6191144	SAL_LA0976AA	29/10/2008	Unknown	*Salmonella* Adjame	3929	421	83564	6.14.14.14.14.14.15
409961	SRR6191363*	SAL_LA0973AA	02/06/2011	Unknown	*Salmonella* Adjame	3929	421	83563	7.16.16.16.16.16.17
409962	SRR6237095	SAL_LA0974AA	26/02/2012	Unknown	*Salmonella* Adjame	3929	421	83562	1.15.15.15.15.15.16
411501	SRR6466751	SAL_LA8147AA	16/03/2011	Unknown	*Salmonella* Adjame	3929	421	88222	1.19.19.21.21.21.22
411502	SRR6466752	SAL_LA8149AA	28/01/2013	Unknown	*Salmonella* Adjame	3929	421	88223	1.19.19.21.22.22.23
416016	SRR6192997	SAL_LA8146AA	09/07/2016	Unknown	*Salmonella* Adjame	3929	421	88221	6.17.20.22.23.23.24

435414	SRR6233939*	SAL_MA5006AA	12/06/2017	Unknown	*Salmonella* Adjame	3929	421	81264	1.8.8.8.8.8.8
202/66	SAL_KA0245AA	SAL_KA0245AA	1966	(From Cote d'Ivoire)	*Salmonella* Adjame	3929	421	88052	1.18.18.20.20.20.21
367320 (Reference Strain)	SRR5583198	SAL_KA8745AA	24/04/2017	Unknown	*Salmonella* Adjame	3929	421	71258	1.1.1.1.1.1.1
MS170178-2	ERR2071995	SAL_KA8199AA	14/06/2017	From Ireland	*Salmonella* Adjame	3929	421	81264	1.8.8.8.8.8.8
MS170185-2	ERR2071997	SAL_KA8197AA	16/06/2017	From Ireland	*Salmonella* Adjame	3929	421	82074	6.17.17.17.17.17.18
SSI-AC209	ERR2234457	SAL_KA0245AA	01/06/2017	From Denmark	*Salmonella* Adjame	4023	421	75355	1.2.2.19.19.19.20

Footnote for Supplementary Table 1 * indicates cases as part of the June-July 2017 outbreak, # Travel to India was stated on the request from but the follow up questionnaire had clarified that this not occurred in the previous 7 days.
